# Social, biological, and programmatic factors linking adolescent pregnancy and early childhood undernutrition: a path analysis of India's 2016 National Family and Health Survey

**DOI:** 10.1016/S2352-4642(19)30110-5

**Published:** 2019-07

**Authors:** Phuong Hong Nguyen, Samuel Scott, Sumanta Neupane, Lan Mai Tran, Purnima Menon

**Affiliations:** a Poverty, Health and Nutrition Division, International Food Policy Research Institute, Washington, DC, USA; b FHI 360, Washington, DC, USA

## Abstract

**Background:**

Adolescent pregnancy and child undernutrition are major social and public health concerns. We aimed to examine associations between adolescent pregnancy and child undernutrition in India, where one in five adolescents live, and one in three of the world's stunted children.

**Methods:**

Data were from India's fourth National Family Health Survey, 2015–16. Primiparous women aged 15–49 years who gave birth between 2010 and 2016 were classified on the basis of age at first birth: 10–19 years (adolescence), 20–24 years (young adulthood), and 25 years or older (adulthood). Primary outcomes were anthropometric measures of offspring undernutrition. Multivariable regression and structural equation models were used to understand the extent to which these measures were linked to adolescent pregnancy and the potential social, biological, and programmatic pathways.

**Findings:**

Of the 60 096 women in the sample, 14 107 (25%) first gave birth during adolescence. Children born to adolescent mothers had lower Z scores for length or height-for-age (mean difference −0·53 SD), weight-for-age (–0·40 SD), and weight-for-length or height (–0·16 SD) than children born to adult mothers. Compared with adult mothers, adolescent mothers were shorter (–1·21 cm, 95% CI −1·78 to −0·65), more likely to be underweight (18 percentage points, 15–21) and anaemic (8 percentage points, 6–11), less likely to access health services (–4 to −15 percentage points), and had poorer complementary feeding practices (–3 to −9 percentage points). Adolescent mothers also had less education (–3·30 years, 95% CI −3·68 to −2·91), less bargaining power (–7 to −15 percentage points), and lived in poorer households (–0·66 SD, 96% CI −0·82 to −0·50) with poorer sanitation (–28 percentage points, −32 to −24). In the path analysis, these intermediate factors predicted child anthropometry, with the strongest links being mother's education (18%), socioeconomic status (13%), and weight (15%).

**Interpretation:**

Children born to adolescent mothers are at risk of being undernourished. Adolescent pregnancy is related to child undernutrition through poor maternal nutritional status, lower education, less health service access, poor complementary feeding practices, and poor living conditions. Policies and programmes to delay pregnancy and promote women's rights could help break the intergenerational cycle of undernutrition through many routes.

**Funding:**

Bill & Melinda Gates Foundation through Partnerships and Opportunities to Strengthen and Harmonize Actions for Nutrition in India, led by the International Food Policy Research Institute.

## Introduction

Adolescence, a period of crucial physical and neuro-maturational changes, is increasingly recognised as a life stage worthy of strategic health investments.^1^ The UN's Sustainable Development Agenda includes specific targets on reducing poverty and hunger, ensuring healthy lives and wellbeing at all ages, empowering women, and achieving equity in education; these targets will not be reached without building the capabilities of adolescents.^2^

South Asia has the largest number of stunted children globally. It is also home to the most adolescents of any global region, and one in five adolescents globally live in India. Among the many health issues that adolescents face, teenage pregnancy is arguably of the greatest consequence due to its effects on the wellbeing of both the mother and child. An estimated 16 million girls aged 15–19 years give birth annually, and 95% of these births occur in low-income and middle-income countries.^3^ Although marriage before the age of 18 years has been illegal in India since 1929—with the law updated as the Prohibition of Child Marriage Act in 2006—girls, especially those from poor rural areas, continue to be married early. Furthermore, owing to societal pressure to consummate the marriage and low sexual reproductive health knowledge, among other factors, 31% of married Indian women gave birth by the age of 18 years in 2016.^4^

**Research in context**

**Evidence before this study**

Childhood stunting is highly prevalent in low-income countries, and children born to adolescent mothers are more likely to be stunted compared with those born to adult mothers. We searched PubMed from database inception to Dec 19, 2018, with filters for English language and human studies and the following search terms: (adolescent OR adolescence OR teenage OR teen) AND (pregnant OR pregnancy) AND (infant OR child) AND (stunting OR stunted). We identified 27 studies that examined the relationship between adolescent pregnancy and childhood stunting. Different studies separately identified relationships related to women's nutrition, access to health services during antenatal, delivery, and early childhood periods, infant and young child feeding, living conditions, and women's education and bargaining power. No studies had empirically examined all seven groups of factors together; the most comprehensive empirical study was from Bangladesh, reporting on four of these seven factors.

**Added value of this study**

To our knowledge, this is the most comprehensive study to date of biological, social, and programmatic factors at multiple levels (individual, household, and health services) that aims to explain the detrimental effects of early childbearing age on child stunting. By use of nationally representative data on more than 60 000 mother-child pairs in India and examination of multiple pathways, we found that adolescent pregnancy is associated with child undernutrition through factors such as poorer maternal nutritional status, lower educational attainment, less access to health services during antenatal or postnatal care and early childhood, suboptimal complementary feeding practices, and poorer living conditions compared with adult pregnancy. Together, these factors accounted for an 11 percentage point higher prevalence of child stunting.

**Implications of all the available evidence**

The findings of this study, together with previous studies, show that adolescent pregnancy is linked to childhood stunting through a wide set of factors. As one of the ten countries with the largest burden of early childbearing in both relative prevalence and absolute number—and the country with the most stunted children—reducing adolescent pregnancy in India can likely help achieve several Sustainable Development Goals related to poverty, health, nutrition, general wellbeing, equity, and education. Policies and programmes to delay childbearing have the potential to help break the intergenerational cycle of poverty and undernutrition through broad effects on multiple determinants of childhood stunting.

The adverse consequences of early childbearing on maternal and child health and wellbeing are far ranging.^5^, ^6^, ^7^, ^8^ Pregnancy and childbirth complications are the leading cause of death among 15–19-year-old girls globally.^9^ Adolescent pregnancy often results in school dropout, affecting young women's education and income.^1^, ^10^ Women's nutritional status is also affected.^11^, ^12^ The prevalence of thinness among Indian women is twice as high in those married before 18 years of age than those married after 24 years of age (33% *vs* 16%).^13^ Furthermore, women who become pregnant as adolescents might not have access to high quality health services during the crucial first 1000-day period,^14^, ^15^ which might have long-term consequences for their children.^5^, ^7^

Despite evidence that pregnancy during adolescence negatively affects maternal and child outcomes, to our knowledge no studies have taken a holistic approach to understanding how early childbearing is linked to child undernutrition. Although studies have examined some potential pathways, few have empirically examined multiple biological, social, and programmatic factors. Understanding these relationships can help to build support for policies and programmes that improve adolescent, maternal, and child health and wellbeing. We aimed to determine to what extent adolescent pregnancy is associated with poor child nutritional outcomes and through which pathways adolescent pregnancy is linked to child undernutrition.

## Methods

### Data sources

This Article uses data from the fourth round of India's National Family Health Survey (NFHS-4)^16^ which is representative at both the state and district levels, gathering data from 601 509 households. To avoid biases associated with parity and birth spacing, we only included primiparous women aged 15–49 years who had given birth in the previous 5 years.^15^ Women were classified on the basis of their age at the time of their first livebirth: 10–19 years (adolescents), 20–24 years (young adults), and 25 years or older (adults). Details of sample selection are provided in the appendix.

### Outcomes

The primary outcomes in this study were child anthropometric measures. Children's weight and length or height measurements were used to derive Z scores by comparing each child's anthropometric measurements with the WHO age-appropriate and sex-appropriate child growth standards.^17^ Three indicators were calculated: length or height-for-age Z score (HAZ), weight-for-age Z score (WAZ), and weight-for-length or height Z score (WHZ). Stunting was defined as a HAZ of less than −2, underweight as a WAZ of less than −2, and wasting as a WHZ of less than −2.^17^

### Framework of determinants

**Figure 1:**
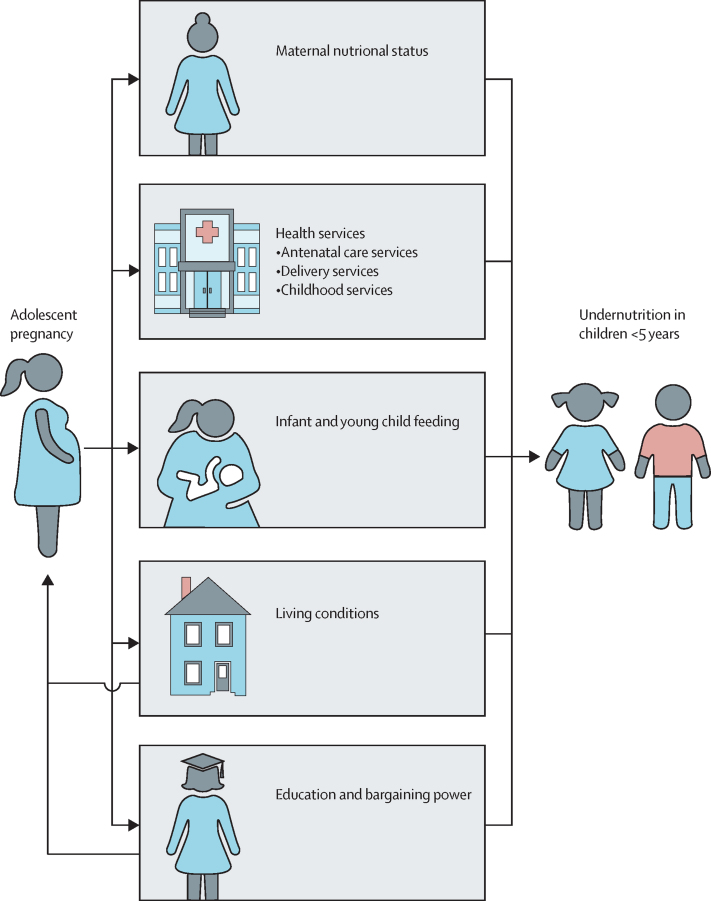
Conceptual framework for linking adolescent pregnancy and early childhood undernutrition

We were not able to find any comprehensive frameworks to explain potential associations between adolescent pregnancy and child undernutrition. Therefore, we developed an evidence-based conceptual framework on the basis of a literature review to guide our path analyses (figure 1). We did a systematic review of the global published literature linking adolescent pregnancy to child stunting. The search details and relevant studies are presented in the appendix. We identified 27 studies published between 1990 and 2018; from these studies we identified five broad groups of factors linking adolescent pregnancy to child undernutrition: maternal nutritional status, education and bargaining power, access to health services, child feeding practices, and living conditions. We used available variables in the NFHS-4 to empirically test the role of these factors, recognising that a few variables reflect the status of the mother at the time of the survey rather than the status at the time of her pregnancy. Details of the indicators used are outlined in the panel. Although we show directionality using arrows in figure 1 for illustrative purposes of early pregnancy possibly leading to child undernutrition, we acknowledge the interplay of factors presented in the framework, such as the bidirectional nature of the links between adolescent pregnancy and maternal education, bargaining power, and living conditions.

Panel **Indicators included in analyses linking adolescent pregnancy to child undernutrition**

**Maternal nutritional status**

Maternal weight and height were used to calculate body-mass index (BMI), with low BMI defined as <18·5 kg/m^2^. Haemoglobin concentrations were measured from capillary blood samples, using a portable HemoCue Hb 201 + analyser. Haemoglobin concentration was adjusted for cigarette smoking and for altitude in areas above 1000 m, and anaemia was defined as a concentration of less than 120 g/L for non-pregnant women.^18^

**Access to nutrition and health services**

*Antenatal care services*

To assess antenatal health services, we included early antenatal care visit (received antenatal care during the first trimester of pregnancy), at least four antenatal care visits, received iron and folic acid supplements, and received deworming during pregnancy.

*Delivery and postnatal health services*

To assess delivery and postnatal health services, we included institutional delivery, skilled birth attendant present during delivery, and postnatal care for mothers during the first 2 days after birth.

*Early childhood health services*

Indicators related to early childhood included growth monitoring, food supplementation (among children ≥6 months of age), full immunisation (among children ≥12 months of age), paediatric iron and folic acid and vitamin A supplementation, and deworming (among children ≥6 months of age).

**Infant and young child feeding**

Infant and young child feeding practices were assessed using the standard WHO indicators,^19^ including early initiation of breastfeeding (the proportion of infants breastfed within 1 h of birth), exclusive breastfeeding (the proportion of infants 0–5·9 months of age who were fed only breast milk), adequate diet (defined as children who consumed at least four of seven food groups in the previous 24 h and age-appropriate meal frequency), and consumption of iron-rich food.^19^

**Living conditions**

A household socioeconomic status index was constructed using principal component analysis, extracting from multiple variables including house and land ownership, housing structure, and ownership of assets and livestock.^20^ Sanitation was captured by whether the household had an improved sanitation facility.

**Education and bargaining power**

Women's status and bargaining power were measured through their education (completed years of education), work for pay in the last 12 months, ownership of money and large assets (land or house), having a say in household decisions (a composite score of decisions on health care, large household purchases, ability to spend the husband's earnings, and whether permission is needed to visit family or relatives), and mobility (a composite score of women's ability to travel alone to the market, to the health facility, and out of the village).

### Statistical analysis

We calculated women's age at first birth as the difference between the birth date of the first-born child and the birth date of the woman. We estimated the proportion of women in the sample who were adolescents, young adults, and adults at the time of their first livebirth. We used survey analysis procedures in Stata version 15 to account for the cluster sampling design and sampling weights in the NFHS-4 in all analyses described here, including these estimates.

To determine the extent to which adolescent pregnancy is associated with poor child nutritional outcomes, we first visualised how child anthropometric outcomes differ among children born to adolescents, young adults, or adult women. We plotted HAZ, WAZ, and WHZ by child age from 0 to 60 months using smoothed polynomial plots. We then compared these outcomes among the groups using multivariable regression models, adjusting for child age, sex, mother's religion, and scheduled caste or tribe (designated groups of historically disadvantaged people in India).

To determine the pathways through which adolescent pregnancy might contribute to child undernutrition, we used multivariable adjusted regression models to examine the association of adolescent pregnancy with the hypothesised linking factors. We used structural equation models, considering all the potential variables in our conceptual framework, to examine the direct and indirect links between adolescent pregnancy and child HAZ. Because sample sizes were smaller for some variables in the NFHS-4, three path models were run. Model 1, using the full sample, included variables for maternal nutritional status, education, access to health services, and living conditions. Model 2 included all variables in Model 1 plus the child feeding variables available only for the subsample of mothers with children 6–24 months of age. Model 3 included all variables in Model 1 plus the women's bargaining power variables in the subsample with available data. The indirect effects, calculated by multiplying coefficients for each path, allowed us to compare the relative strength of each path.

As a robustness check for all models, we also added state fixed effects to account for state-level heterogeneity in externalities that might affect maternal and child health, such as state-specific programmes and policies.

### Role of the funding source

The funder of the study had no role in study design, data collection, data analysis, data interpretation, or writing of the report. All authors had access to all the data and had final responsibility for the decision to submit.

## Results

Of 60 096 women aged 15–49 included in the final sample (appendix), 14 107 (25·1%) first gave birth during adolescence, 31 475 (52·3%) during young adulthood, and 14 514 (23·2%) during adulthood. As expected, women who first gave birth during adolescence got married earlier (mean age at marriage 16·4 years of age, SD 1·67) compared with those who first gave birth as young adults (19·7, 2·14) or adults (24·3, 3·87; appendix). A greater proportion of women who first gave birth during adolescence lived in rural areas and belonged to a disadvantaged group compared with women who first gave birth as adults (appendix). The age and sex ratio of firstborn children was similar across groups (appendix).

**Figure 2:**
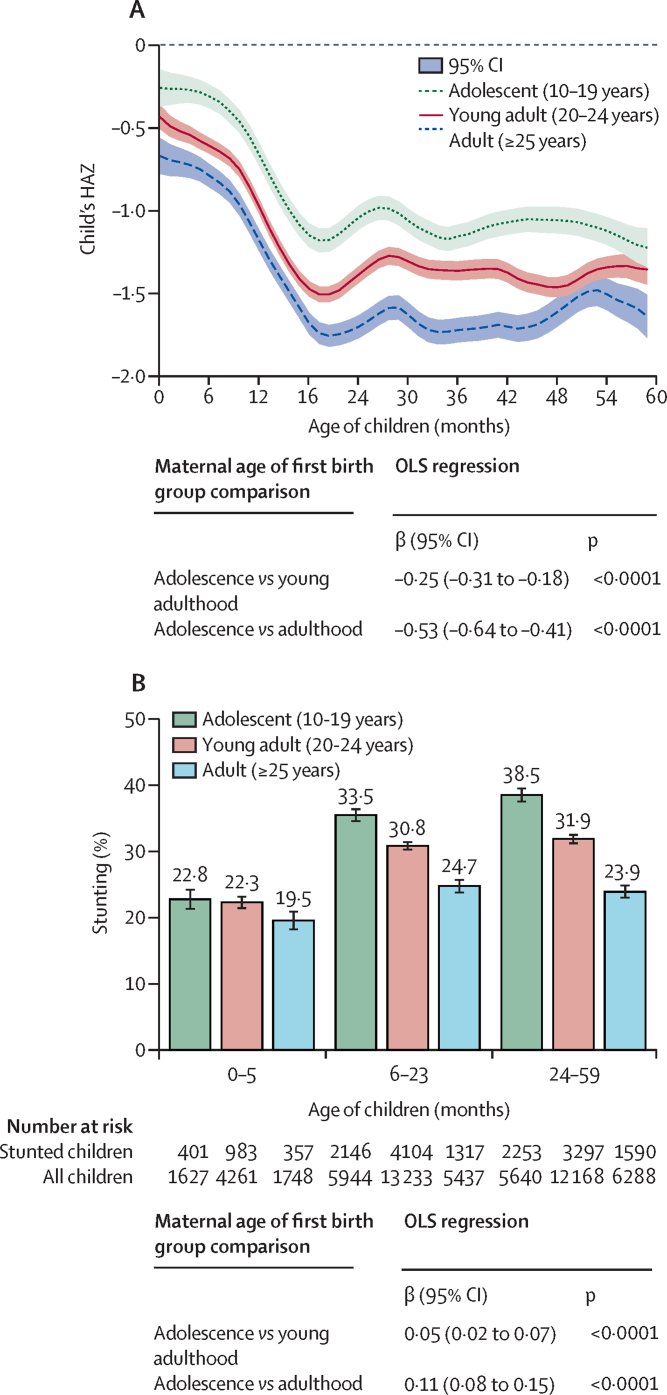
HAZ score and prevalence of stunting for first-born children by child's age and mother's age at first birth in India, 2016 (A) HAZ score. Curves are smooth local polynomials with 95% CIs. (B) Stunting. Adjusted coefficient (95% CI) for models are shown below each panel in the figure. OLS regression models were adjusted for child age, sex, maternal religion, and caste fixed effects and controlled for the cluster sampling design and sampling weights used in the survey. Data are from India's fourth National Family Health Survey (NFHS-4, 2016).^16^ HAZ=Length or height-for-age Z score. OLS=ordinary least squares.

At all ages between 0 and 60 months, children born to adolescent mothers had lower HAZ and WAZ than children born to young adults or adult women (figure 2; appendix). For WHZ, a similar trend was observed in children older than 6 months, with slightly smaller group differences compared with HAZ and WAZ (appendix).

The multivariable regression confirmed that children born to adolescent mothers were 0·25 SD (95% CI −0·31 to −0·18) shorter for their age on average and were 5 percentage points (95% CI 2–7) more likely to be stunted than children born to young adults (figure 2). These differences were more prominent when comparing children born to adolescents with those born to adults (β=–0·53 [95% CI −0·64 to −0·41] for height-for-age Z score and 11 percentage points [8–15] for stunting). Compared with children born to adult mothers, children born to adolescent mothers also had lower WAZ (β=–0·40, 95% CI −0·47 to −0·32), lower WHZ (β=–0·16, −0·23 to −0·08), and higher prevalence of underweight (10 percentage points, 7–12) but not wasting (appendix).

Adolescent pregnancy was negatively associated with maternal nutritional status (table). Compared with women who first gave birth as adults, women who first gave birth during adolescence were shorter, weighed less, had lower haemoglobin, had lower body-mass index, and had a higher prevalence of underweight and anaemia (table).

**Table:** Association between maternal age at first birth and factors related to child nutritional status in India, 2016

	**Total women**	**First birth during adolescence (10–19 years)**		**First birth during young adulthood (20–24 years)**		**First birth during adulthood (≥25 years)**		Adolescence *vs* young adulthood*		Adolescence *vs* adulthood*	
		Number of women	n (%) or mean	Number of women	n (%) or mean	Number of women	n (%) or mean	β (95%CI)	p value	β (95%CI)	p value
**Maternal nutritional status**											
Height, cm	51 281	11 576	151·47 (5·54)	26 747	152·15 (5·82)	12 958	152·79 (6·12)	−0·63 (−1·09 to −0·18)	0·0073	−1·21 (−1·78 to −0·65)	<0·0001
Weight, kg	51 342	11 588	46·64 (8·27)	26 772	49·03 (9·52)	12 982	53·68 (11·34)	−2·29 (−2·91 to 1·67)	<0·0001	−6·81 (−7·71 to −5·91)	<0·0001
BMI, kg/m^2^	51 242	11 568	20·30 (3·25)	26 725	21·15 (3·76)	12 949	22·96 (4·49)	−0·82 (−1·02 to −0·62)	<0·0001	−2·59 (−2·93 to −2·25)	<0·0001
BMI <18·5 kg/m^2^	51 242	11 568	3777 (32·56%)	26 725	6581 (25·19%)	12 949	1756 (13·88%)	0·07 (0·05 to 0·09)	<0·0001	0·18 (0·15 to 0·21)	<0·0001
Haemoglobin, g/dL	51 076	11 520	11·55 (1·46)	26 663	11·65 (1·54)	12 893	11·83 (1·58)	−0·09 (−0·14 to −0·04)	0·0010	−0·26 (−0·35 to −0·17)	<0·0001
Anaemia	51 076	11 520	6512 (58·02%)	26 663	14 420 (54·83%)	12 893	6224 (48·97%)	0·03 (0·01 to 0·04)	0·0008	0·08 (0·06 to 0·11)	<0·0001
**Access to antenatal care services during pregnancy**											
Early antenatal care	60 012	14 090	8296 (60·32%)	31 426	20 876 (67·54%)	14 496	10 494 (72·55%)	−0·07 (−0·09 to −0·04)	<0·0001	−0·12 (−0·16 to −0·07)	<0·0001
At least 4 antenatal care visits	60 012	14 090	6893 (56·66%)	31 426	17 723 (60·57%)	14 496	9917 (72·05%)	−0·04 (−0·11 to 0·03)	0·29	−0·15 (−0·26 to −0·04)	0·0068
Received iron and folic acid	59 920	14 059	11 122 (80·56%)	31 389	26 077 (83·73%)	14 472	12 605 (88·35%)	−0·03 (−0·07 to 0·01)	0·098	−0·08 (−0·13 to −0·03)	0·0039
Received deworming	59 442	13 961	2253 (18·39%)	31 153	5509 (20·27%)	14 328	2797 (23·58%)	−0·02 (−0·04 to 0·01)	0·14	−0·05 (−0·08 to −0·02)	0·0013
**Access to delivery and postpartum services**											
Institutional delivery	60 001	14 089	11 815 (86·95%)	31 419	28 058 (91·62%)	14 493	13 501 (95·61%)	−0·04 (−0·05 to −0·03)	<0·0001	−0·08 (−0·11 to −0·05)	<0·0001
Skilled birth attendant at delivery	60 096	14 107	12 045 (88·28%)	31 475	28 377 (92·11%)	14 514	13 556 (95·18%)	−0·03 (−0·05 to −0·02)	<0·0001	−0·06 (−0·09 to −0·04)	<0·0001
Postnatal care for mothers	60 096	14 107	8756 (65·65%)	31 475	21 647 (70·80%)	14 514	11 002 (77·27%)	−0·05 (−0·08 to −0·02)	0·0011	−0·11 (−0·16 to −0·06)	<0·0001
**Access to services during childhood**											
Growth monitoring	60 096	14 107	6563 (50·43%)	31 475	14 319 (45·78%)	14 514	5518 (38·59%)	0·04 (−0·02 to 0·11)	0·21	0·11 (0·01 to 0·21)	0·025
Food supplementation	60 096	14 107	7863 (58·69%)	31 475	16 716 (52·05%)	14 514	6675 (43·35%)	0·06 (−0·01 to 0·12)	0·056	0·14 (0·05 to 0·23)	0·0025
Full immunisation†	41 667	9990	6082 (64·69%)	21 488	14 381 (67·21%)	10 189	7185 (70·74%)	−0·02 (−0·08 to 0·03)	0·39	−0·06 (−0·13 to 0·00)	0·097
Vitamin A‡	50 978	12 126	6886 (60·46%)	26 506	15 769 (62·14%)	12 346	7803 (66·68%)	−0·02 (−0·05 to 0·02)	0·31	−0·06 (−0·08 to −0·04)	<0·0001
Child iron and folic acid‡	50 669	12 042	2945 (27·04%)	26 350	6978 (28·55%)	12 277	3424 (31·59%)	−0·02 (−0·04 to 0·01)	0·19	−0·04 (−0·07 to −0·02)	0·0005
Child deworming‡	50 647	12 030	3666 (35·26%)	26 351	8224 (33·76%)	12 266	4279 (37·61%)	0·01 (−0·04 to 0·07)	0·65	−0·02 (−0·09 to 0·06)	0·69
**Infant and young child feeding practices**											
Early initiation of breastfeeding§	33 313	7831	3512 (43·59%)	17 999	7595 (42·49%)	7483	3352 (42·82%)	0·01 (−0·02 to 0·04)	0·60	0·01 (−0·03 to 0·03)	0·85
Exclusive breastfeeding¶	8419	1777	1137 (63·38%)	4661	2845 (60·69%)	1981	1246 (59·50%)	0·03 (−0·01 to 0·07)	0·13	0·04 (−0·01 to 0·08)	0·10
Adequate diet‖	25 494	6144	529 (8·69%)	13 673	1209 (9·13%)	5677	653 (11·15%)	−0·01 (−0·02 to 0·01)	0·30	−0·03 (−0·05 to −0·01)	0·049
Consumption of iron-rich food‖	25 871	6257	1430 (23·18%)	13 847	3251 (23·92%)	5767	1966 (32·75%)	−0·01 (−0·04 to 0·02)	0·57	−0·09 (−0·13 to −0·06)	<0·0001
**Living conditions**											
Socioeconomic status index	60 096	14 107	−0·32 (0·87)	31 475	0·03 (0·91)	14 514	0·39 (0·87)	−0·33 (−0·41 to −0·25)	<0·0001	−0·66 (−0·82 to −0·50)	<0·0001
Improved sanitation facility	60 096	14 107	5509 (38·80%)	31 475	15 979 (51·93%)	14 514	9678 (68·58%)	−0·12 (−0·16 to −0·09)	<0·0001	−0·28 (−0·32 to −0·24)	<0·0001
**Education and bargaining power**											
Education, years	60 096	14 107	7·27 (3·96)	31 475	8·96 (4·84)	14 514	10·81 (5·48)	−1·54 (−1·83 to −1·26)	<0·0001	−3·30 (−3·68 to −2·91)	<0·0001
Works for pay	10 502	2348	411 (15·36%)	5409	852 (13·78%)	2745	598 (21·37%)	0·01 (−0·01 to 0·03)	0·37	−0·07 (−0·12 to −0·01)	0·015
Ownership of money	10 340	2302	755 (35·82%)	5341	2093 (40·43%)	2697	1329 (49·95%)	−0·05 (−0·10 to 0·01)	0·084	−0·14 (−0·20 to −0·08)	<0·0001
Ownership of land or house	10 561	2365	919 (32·85%)	5435	2072 (34·76%)	2761	1087 (36·59%)	−0·02 (−0·06 to 0·03)	0·39	−0·04 (−0·11 to 0·03)	0·26
Say in household decisions	10 340	2302	0·66 (0·38)	5341	0·69 (0·39)	2697	0·74 (0·39)	−0·03 (−0·06 to 0·00)	0·040	−0·08 (−0·12 to −0·04)	0·0007
Mobility	10 561	2365	0·38 (0·42)	5435	0·41 (0·45)	2761	0·53 (0·47)	−0·03 (−0·11 to 0·04)	0·38	−0·15 (−0·24 to −0·07)	0·0008

Data are from India's fourth National Family Health Survey^16^ and adjusted to account for cluster sampling design and sampling weights. BMI=body-mass index.

* Maternal outcomes adjusted for maternal caste and religion, and child outcomes adjusted for child age, gender, maternal caste, and religion fixed effects.

† Children aged 12–59 months.

‡ Children aged >6 months.

§ Children aged 0–23 months.

¶ Children aged <6 months.

‖ Children aged 6–23 months.

Women who first gave birth during adolescence had poorer access to and use of antenatal care services than did women who first gave birth during adulthood (table). Adolescent pregnancy was also associated with less service use during delivery and early postpartum and poorer access to services during early childhood (table). Adolescent pregnancy was positively associated with child growth monitoring and food supplementation (table), both interventions delivered by India's national Integrated Child Development Services programme.

Adolescent pregnancy was negatively associated with complementary feeding practices (table). No differences were found between adolescents, young adult, and adult mothers for breastfeeding.

Adolescent pregnancy was negatively associated with optimal living conditions, women's education, and most bargaining power indicators except for work for pay and ownership of land or house. Compared with adult mothers, women who gave birth during adolescence were more likely to live in a household with lower socioeconomic status and poorer sanitation (table). Women who first gave birth during adolescence had 3·30 (95% CI −3·68 to −2·91) fewer years of education, were less likely to work for pay, had lower power in ownership of money, less say in household decisions, and less freedom of mobility (table).

**Figure 3:**
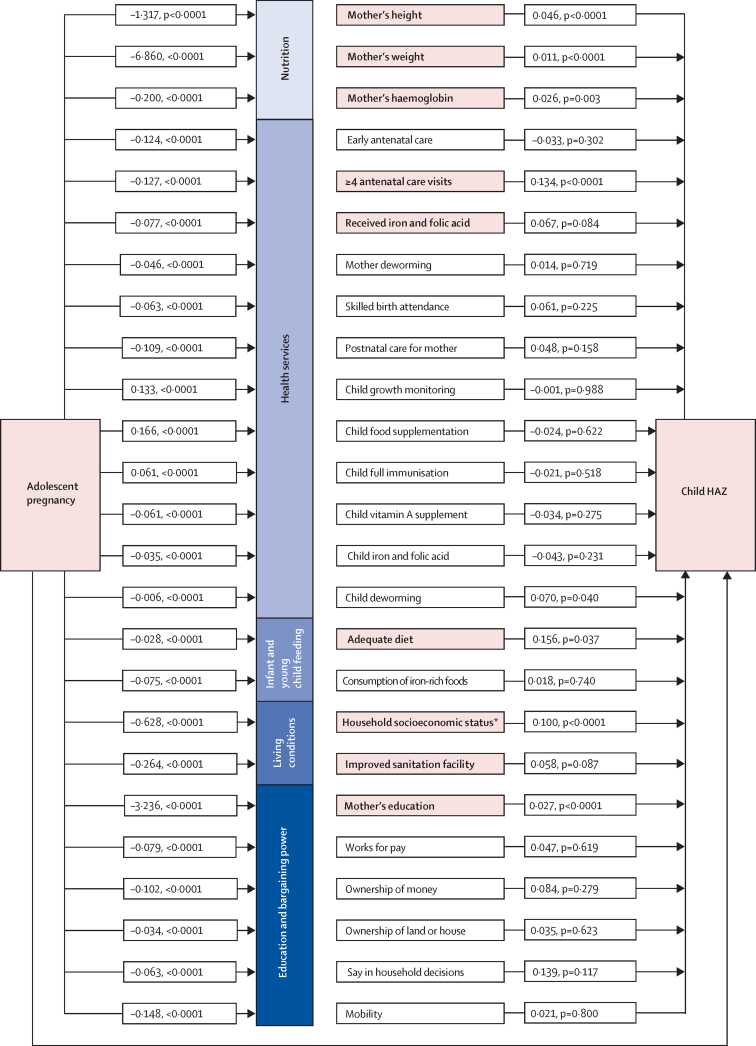
Pathways from adolescent pregnancy to child undernutrition through maternal nutrition, health services, infant and young child feeding practices, living conditions, women's education, and bargaining power Bold font and shading for the mediating variables indicates that the variable was important in mediating the association between adolescent pregnancy and child height-for-age Z score (p<0·1 for both steps). Numbers in the paths are regression coefficients. Figure presents paths from the three path models that were run. Model 1 included variables for maternal nutritional status, access to health and nutrition services, living conditions, and women's education. Model 2 included all variables in model 1 plus the child feeding variables shown, in the subsample of mothers with children 6–24 months of age (n=25 494). Model 3 included all variables in model 1 plus the bargaining power variables shown, in the subsample with data available (n=10 340). All models adjusted for maternal caste and religion and child age and sex and controlled for the cluster sampling design and sampling weights used in the survey. Coefficients shown in the figure are from Model 1, except for those for child feeding (from Model 2) and bargaining power (from Model 3). HAZ=Length or height-for-age Z score. *Constructed from principal components analysis of asset ownership.

In the full path model considering all available factors, we found evidence of strong links between adolescent pregnancy and child undernutrition through maternal nutritional status (proxied by height, weight, and haemoglobin), access to antenatal care services (at least four antenatal care visits and receiving iron and folic acid during pregnancy), women's education, living conditions (household socioeconomic status and sanitation), and adequate child diet (figure 3). The indirect path through these factors accounted for 68·5% of the relationship between adolescent pregnancy on child HAZ (ie, the 0·53 SD difference). Overall, after considering relative indirect effects for all pathways, the strongest links between adolescent pregnancy on child HAZ worked through education (which accounted for 18% of the diffence), socioeconomic status (13% of the difference), and maternal weight (15% of the difference).

## Discussion

As the global adolescent population grows, reducing early childbearing is crucial to achieving the Sustainable Development Goals related to poverty, health, nutrition, general wellbeing, equity, and education. By use of data from India's largest nationally representative health survey in 2015–16, NFHS-4,^16^ we aimed to understand how early childbearing relates to child undernutrition through a set of social, biological, and programmatic factors related to adolescent pregnancy. We found that stunting and underweight were 11 percentage points more prevalent in children born to adolescent mothers compared with children born to adult mothers. Our path analyses showed that adolescent pregnancy is associated with poorer maternal nutritional status, lower educational attainment, lower likelihood of accessing antenatal health services, poorer complementary feeding practices, and poorer living conditions, all of which were also associated with child stunting.

Compared with women who first gave birth as adults, women who were mothers in adolescence were shorter and thinner, and these maternal nutritional insults were associated with lower length or height and weight in their children. Although height and weight in our study were measured at the time of survey, up to 5 years after the mother's first birth, other evidence provides insights into the biological link between maternal and child anthropometry. For example, in a study done in Bangladesh, pregnancy and lactation ceased linear growth and resulted in weight loss and depletion of fat and lean body mass in adolescent girls (aged 12–19 years),^12^ probably due to concurrent competition for nutrients between the mother, who is still growing, and fetus. Similarly, a multinomial regression analysis using the third round of India's NFHS (2005–06) found a large adverse effect of early marriage and childbearing on thinness in women.^13^ In our study, adolescent mothers were more likely to be anaemic compared with adult mothers, and anaemia was associated with reduced child growth. However, these findings should be interpreted with caution as a mother's anaemia and weight status might not reflect her earlier status at the time of pregnancy. However, current maternal height could be more closely associated with height at the time of pregnancy, because adolescent height growth in girls usually peaks before menarche.

Adolescent mothers had about 3 fewer years of education compared with adult mothers. They were also less likely to work for pay, to have money they could spend, to have a say in household decisions, and to be able to travel without permission. Among these factors, only education was related to child HAZ; the path was one of the strongest observed, with the effect of adolescent pregnancy on mother's education accounting for an 18% difference in child HAZ. School-aged girls (aged 11–17 years) who marry and bear children are more likely to discontinue their education compared with those who do not marry or become pregnant during school.^21^, ^22^ Furthermore, maternal education is known to be a strong determinant of many child outcomes, including linear growth.^23^ Women's bargaining power might still be an important determinant of child nutrition; however the effects might have been outweighed by education in our multivariable model.

Use of health services during antenatal period was an important link between adolescent pregnancy and child nutrition. Our results match findings from another study^15^ in west Africa showing that adolescent (aged 10–19 years) mothers seek care later, make fewer visits during pregnancy, and receive fewer components of care than older first-time mothers. Our work extends these findings by examining implications during delivery and postnatal periods as well as at the child level. Whereas most antenatal health service factors were important in our path models, we found that receipt of health services during postnatal and early childhood periods had less of a role. Although adolescent pregnancy was associated with poorer access to services during the postpartum and early childhood periods, these indicators were not significantly associated with child HAZ in our multivariable regression model after controlling for maternal education and living conditions.

Complementary feeding practices were identified as important in the relationship between adolescent pregnancy and child nutrition. In our study, children born to adolescent mothers were less likely to achieve adequate diet and consume iron-rich foods. Although the reasons for these findings are not possible to ascertain from the available survey data, poor complementary feeding among adolescent mothers could stem from poorer living conditions (which affect access to adequate diets), lower access to information through lower use of health services, both of which we find in our study, or a reduced cognitive or emotional ability to manage the demands of a young infant.^24^ Adequate diet has been found to have strong association with child growth, both from global^25^ and Indian data.^26^

Socioeconomic status and improved sanitation at the household level were key links between adolescent pregnancy and poor child nutrition. Adolescent pregnancy could cause poverty over multiple generations, but it is more likely that adolescent pregnancy would occur in high rather than low poverty contexts in the short term. Adolescent pregnancy might perpetuate the cycle of poverty, because women who bear children early are more likely to discontinue education and, thus, have lower earning potential.

Previous published literature examining the consequences of adolescent childbearing has focused on direct mother and child effects but has not linked teen pregnancy to child nutrition outcomes through a wide set of factors faced by adolescents. Our study is the first to consider multiple pathways at multiple levels (social, biological, and programmatic factors) to explain the detrimental effects of early childbearing age on child undernutrition. Our findings are strengthened by the fact that we only included primiparous mothers. Although we sacrificed sample size using this approach, we felt that it was important given the strong confounding effect of child birth order and birth spacing on child nutritional status.^15^, ^27^

Our study contains some methodological limitations that deserve consideration. The cross-sectional design reduces causal inference. For example, becoming pregnant early might lead to reduced education or wealth; however, a woman from a poor background and lower education might be more likely to become pregnant early. Thus, longitudinal data are needed to establish causal relationships. Recall bias in women with older children might have affected their responses to questions about prepregnancy health service use, but this bias is probably uniform across groups, because the child age distributions were similar for children born to adolescents, young adults, and adults. Current poor health and living conditions might reflect an equally poor situation for the mother in past years, and we accept that this history of combined insults probably leads to the child's current anthropometric state. Although women's bargaining power variables were only collected for a random subsample in NFHS-4, the characterisitics of this subsample was similar to the full sample (appendix). Therefore, we believe that the analysed sample provides unbiased estimates for the bargaining power pathway. We did not examine child marriage among boys, which was about the same as the percentage of girls married at less than 18 years of age.^16^ Future research should explore whether the effects of early childbearing on child undernutrition work through paternal pathways, especially in light of transitioning gender roles and efforts to shift the focus of paediatric care from the mother-child dyad to the mother-father-child triad.^28^

Interventions to increase age at first marriage, age at first birth, and girl's education are a promising approach to also improve maternal and child nutrition. A review of interventions to prevent child marriage in low-income and middle-income countries found that six of 11 high-quality studies showed some positive effect on decreasing child marriage (marriage before 18 years of age) or increasing age at marriage—interventions included unconditional cash transfers, cash transfers conditional on school enrollment or attendance, school vouchers, life-skills curriculum, and livelihood training.^29^ In the past 25 years, the Government of India has piloted different cash transfers conditional on education, with complementary programming meant to encourage investment in girls’ human capital, and several adolescent health programmes are ongoing under different ministries in India.^30^ We acknowledge the strong cultural barriers to efforts to delaying early marriage in this context; however, the declines already seen in the past decade suggest that further investments in appropriate interventions targeting young people, including men, can contribute to further declines in early marriage and early childbearing in India. The high variability in adolescent pregnancy across states and districts suggests that subnational policies and programmes targeting early marriage and early childbearing might well be needed to address differences in cultural practices and other conditions affecting early marriage and early childbearing.

Investments in adolescents have a high benefit-to-cost ratio and yield a triple dividend on the health and wellbeing of adolescents, adults, and the next generation.^2^ Early marriage and childbearing are at the root of many other adolescent issues, because these practices have adverse effects on health, education, and future employment, all of which have intergenerational consequences. The evidence presented here suggests that adolescent pregnancy also has substantial consequences for India's undernutrition burden, operating through multiple factors. Continued investments, especially if focused in areas and communities with high levels of early marriage and childbearing, could have long-ranging positive effects.
