# Supplementary material for: Social, biological, and programmatic factors linking adolescent pregnancy and early childhood undernutrition: a path analysis of India's 2016 National Family and Health Survey
Source: Lancet Child Adolesc Health. 2019 Jul;3(7):463–73. doi: 10.1016/S2352-4642(19)30110-5 (PMC6558962; doi:10.1016/S2352-4642(19)30110-5)
Supplement: Supplementary appendix [file mmc1.pdf]

THE LANCET
Child & Adolescent Health

Supplementary appendix

This appendix formed part of the original submission and has been peer reviewed. We post it as supplied by the authors.

Supplement to: Nguyen PH, Scott S, Neupane S, Tran LM, Menon P. Social, biological, and programmatic factors linking adolescent pregnancy and early childhood undernutrition: a path analysis of India's 2016 National Family and Health Survey. *Lancet Child Adolesc Health* 2019; published online May 15. http://dx.doi.org/10.1016/S2352-4642(19)30110-5.

Supplemental Table 1. Systematic literature review of evidence for linkage between adolescent pregnancy and child undernutrition*

**Author, year**	**Country**	**Outcomes examined**							
		Child undernutrition	Education & bargaining power	Maternal nutrition	Access to ANC services	Access to delivery services	Access to services during childhood	Infant and young child feeding	Living Conditions
Kane *et al.*, 2018[1]	US	x							
Sarker *et al.*, 2018[2]	Bangladesh		x						x
Marphatia *et al.*, 2017[3]	Bangladesh, India, Nepal, and Pakistan	x	x	x	x	x			x
Chari *et al.*, 2017[4]	India	x	x				x		
Yussif *et al.*, 2017[5]	Ghana	x							
Nguyen *et al.*, 2017[6]	Bangladesh	x	x	x					
Islam *et al.*, 2017[7]	Bangladesh		x						x
Delprato *et al.*, 2017[8]	Sub-Saharan Africa and Southwest Asia		x		x	x	x		
Owolabi *et al.*, 2017[9]	13 west African countries				x				
Acharya *et al.*, 2017[10]	Nepal					x			
Mombo-Ngoma *et al.*, 2016[11]	Benin, Gabon, Mozambique, Tanzania	x	x	x	x				
Vir, 2016[12]	South Asia	x	x	x	x	x	x	x	x
Traisrisilp *et al.*, 2015[13]	Thailand	x							
Fall *et al.*, 2015[14]	Brazil, Guatemala, India, Philippines, and South Africa	x							
Goli *et al.*, 2015[15]	India			x					
Ganchimeg *et al.*, 2014[16]	29 countries in Africa, Latin America, Asia and the Middle East.	x							
Fink *et al.*, 2014[17]	61 LMICs	x		x					x
Godha *et al.*, 2013[18]	India, Bangladesh, Nepal, and Pakistan				x	x			
Prakash *et al.*, 2011[19]	India	x	x	x					x
Bakker *et al.*, 2011[20]	The Netherlands	x	x	x					
Rah *et al.*, 2008[21]	Bangladesh			x					
Abdullah *et al.*, 2007[22]	Bangladesh	x	x	x			x		x

Chandra *et al*., 2002[23]	US	x	x	
Motil *et al*., 1997[24]	US			x
Verrier et al., 1993[25]	US	x		
Fraser *et al,* 1995[26]	US	x		
Keterlinus *et al*., 1990[27]	US	x		

*The search was conducted in PubMed between December 15 and 19, 2018. Search terms included (adolescent OR adolescence OR teenage OR teen) AND (pregnant OR pregnancy) AND (infant OR child) AND (stunting OR stunted); English language and Human study filters were applied; the search yielded 1170 items, which were screened for relevance. Among the relevant items, references were also examined for additional relevant studies.

1. Kane JB, Harris KM, Siega-Riz AM. Intergenerational pathways linking maternal early life adversity to offspring birthweight. *Soc Sci Med* 2018; **207**: 89-96.
2. Sarker AR, Sheikh N, Mahumud RA, Sultana M. Determinants of adolescent maternal healthcare utilization in Bangladesh. *Public Health* 2018; **157**: 94-103.
3. Marphatia AA, Ambale GS, Reid AM. Women's Marriage Age Matters for Public Health: A Review of the Broader Health and Social Implications in South Asia. *Front Public Health* 2017; **5**: 269.
4. Chari AV, Heath R, Maertens A, Fatima F. The causal effect of maternal age at marriage on child wellbeing: Evidence from India. *Journal of Development Economics* 2017; **127**: 42-55.
5. Yussif AS, Lassey A, Ganyaglo GY, Kantelhardt EJ, Kielstein H. The long-term effects of adolescent pregnancies in a community in Northern Ghana on subsequent pregnancies and births of the young mothers. *Reprod Health* 2017; **14**(1): 178.
6. Nguyen PH, Sanghvi T, Tran LM, et al. The nutrition and health risks faced by pregnant adolescents: Insights from a cross-sectional study in Bangladesh. *PLoS One* 2017; **12**(6): e0178878.
7. Islam MM, Islam MK, Hasan MS, Hossain MB. Adolescent motherhood in Bangladesh: Trends and determinants. *PLoS One* 2017; **12**(11): e0188294.
8. Delprato M, Akyeampong K. The Effect of Early Marriage Timing on Women's and Children's Health in Sub-Saharan Africa and Southwest Asia. *Ann Glob Health* 2017; **83**(3-4): 557-67.
9. Owolabi OO, Wong KLM, Dennis ML, et al. Comparing the use and content of antenatal care in adolescent and older first-time mothers in 13 countries of west Africa: a cross-sectional analysis of Demographic and Health Surveys. *Lancet Child Adolesc Health* 2017; **1**(3): 203-12.
10. Acharya P, Adhikari TB, Neupane D, Thapa K, Bhandari PM. Correlates of institutional deliveries among teenage and non-teenage mothers in Nepal. *PLoS One* 2017; **12**(10): e0185667.
11. Mombo-Ngoma G, Mackanga JR, Gonzalez R, et al. Young adolescent girls are at high risk for adverse pregnancy outcomes in sub-Saharan Africa: an observational multicountry study. *BMJ Open* 2016; **6**(6): e011783.
12. Vir SC. Improving women's nutrition imperative for rapid reduction of childhood stunting in South Asia: coupling of nutrition specific interventions with nutrition sensitive measures essential. *Maternal & child nutrition* 2016; **12 Suppl 1**: 72-90.
13. Traisrisilp K, Jaiprom J, Luewan S, Tongsong T. Pregnancy outcomes among mothers aged 15 years or less. *J Obstet Gynaecol Res* 2015; **41**(11): 1726-31.
14. Fall CH, Sachdev HS, Osmond C, et al. Association between maternal age at childbirth and child and adult outcomes in the offspring: a prospective study in five low-income and middle-income countries (COHORTS collaboration). *Lancet Glob Health* 2015; **3**(7): e366-77.
15. Goli S, Rammohan A, Singh D. The Effect of Early Marriages and Early Childbearing on Women's Nutritional Status in India. *Matern Child Health J* 2015; **19**(8): 1864-80.
16. Ganchimeg T, Ota E, Morisaki N, et al. Pregnancy and childbirth outcomes among adolescent mothers: a World Health Organization multicountry study. *BJOG* 2014; **121 Suppl 1**: 40-8.
17. Fink G, Sudfeld CR, Danaei G, Ezzati M, Fawzi WW. Scaling-up access to family planning may improve linear growth and child development in low and middle income countries. *PLoS One* 2014; **9**(7): e102391.

18. Godha D, Hotchkiss DR, Gage AJ. Association between child marriage and reproductive health outcomes and service utilization: a multi-country study from South Asia. *J Adolesc Health* 2013; **52**(5): 552-8.
19. Prakash R, Singh A, Pathak PK, Parasuraman S. Early marriage, poor reproductive health status of mother and child well-being in India. *J Fam Plann Reprod Health Care* 2011; **37**(3): 136-45.
20. Bakker R, Steegers EA, Biharie AA, Mackenbach JP, Hofman A, Jaddoe VW. Explaining differences in birth outcomes in relation to maternal age: the Generation R Study. *BJOG* 2011; **118**(4): 500-9.
21. Rah JH, Christian P, Shamim AA, Arju UT, Labrique AB, Rashid M. Pregnancy and lactation hinder growth and nutritional status of adolescent girls in rural Bangladesh. *The Journal of nutrition* 2008; **138**(8): 1505-11.
22. Abdullah K, Malek MA, Faruque AS, Salam MA, Ahmed T. Health and nutritional status of children of adolescent mothers: experience from a diarrhoeal disease hospital in Bangladesh. *Acta Paediatr* 2007; **96**(3): 396-400.
23. Chandra PC, Schiavello HJ, Ravi B, Weinstein AG, Hook FB. Pregnancy outcomes in urban teenagers. *Int J Gynaecol Obstet* 2002; **79**(2): 117-22.
24. Motil KJ, Kertz B, Thotathuchery M. Lactational performance of adolescent mothers shows preliminary differences from that of adult women. *J Adolesc Health* 1997; **20**(6): 442-9.
25. Verrier M, Spears W, Ying J, Kerr GR. Patterns of birth weight in relation to gestational age, maternal age, parity, and prenatal care in Texas' triethnic population, 1984 through 1986. *Tex Med* 1993; **89**(12): 51-6.
26. Fraser AM, Brockert JE, Ward RH. Association of young maternal age with adverse reproductive outcomes. *N Engl J Med* 1995; **332**(17): 1113-7.
27. Ketterlinus RD, Henderson SH, Lamb ME. Maternal age, sociodemographics, prenatal health and behavior: influences on neonatal risk status. *J Adolesc Health Care* 1990; **11**(5): 423-31.

Supplemental Table 2. Household, maternal and child characteristics of primiparous Indian women aged 15-49 years by age at first birth.

	**First birth during adolescence (10-19 y)**		**First birth during young adulthood (20-24 y)**		**First birth during adulthood (25 -49 y)**	
**Household**	*Total women*	*N (%) or mean*	*Total women*	*N (%) or mean*	*Total women*	*N (%) or mean*
Household size, n	14,107	6·02	31,475	5·91	14,514	5·50
Place of residence (rural)	14,107	11,243 (76·60)	31,475	22,824 (67·02)	14,514	8,584 (48·95)
Religion, %						
Hindu	14,107	10,414 (77·67)	31,475	24,174 (80·94)	14,514	10,162(80·41)
Muslim	14,107	2,284 (18·41)	31,475	4,003 (13·54)	14,514	1,724 (10·28)
Other	14,107	1,409 (3·92)	31,475	3,298 (5·52)	14,514	2,628 (9·31)
**Mother**						
Age, y	14,107	20·07	31,475	23·60	14,514	29·59
Age at marriage, y	13,718	16·41	30,855	19·68	14,128	24·31
Age at first birth, y	14,107	18·08	31,475	21·76	14,514	27·60
Caste category, %						
Scheduled caste	14,107	2,722 (22·23)	31,475	5,605 (20·30)	14,514	2,072 (17·80)
Scheduled tribe	14,107	2,946 (12·14)	31,475	5,263 (9·21)	14,514	2,723 (7·13)
Other backward class	14,107	5,309 (38·70)	31,475	12,828 (43·87)	14,514	4,751 (40·11)
Occupation, %						
Not in work force	2,348	1,937 (84·64)	5,409	4,557 (86·22)	2,745	2,147 (78·63)
Agriculture or housewife	2,348	299 (10·27)	5,409	530 (8·10)	2,745	258 (8·74)
Other	2,348	112 (5·08)	5,409	322 (5·68)	2,745	340 (12·63)
**Child**						
Age, mo	13,903	22·87	31,167	22·40	14,327	24·32
Male child, %	14,107	7,459 (52·65)	31,475	16,863 (53·87)	14,514	7,623 (52·86)

Data are from India’s fourth National Family Health Survey (NFHS4, 2016)

Supplemental Figure 1. Study sample diagram

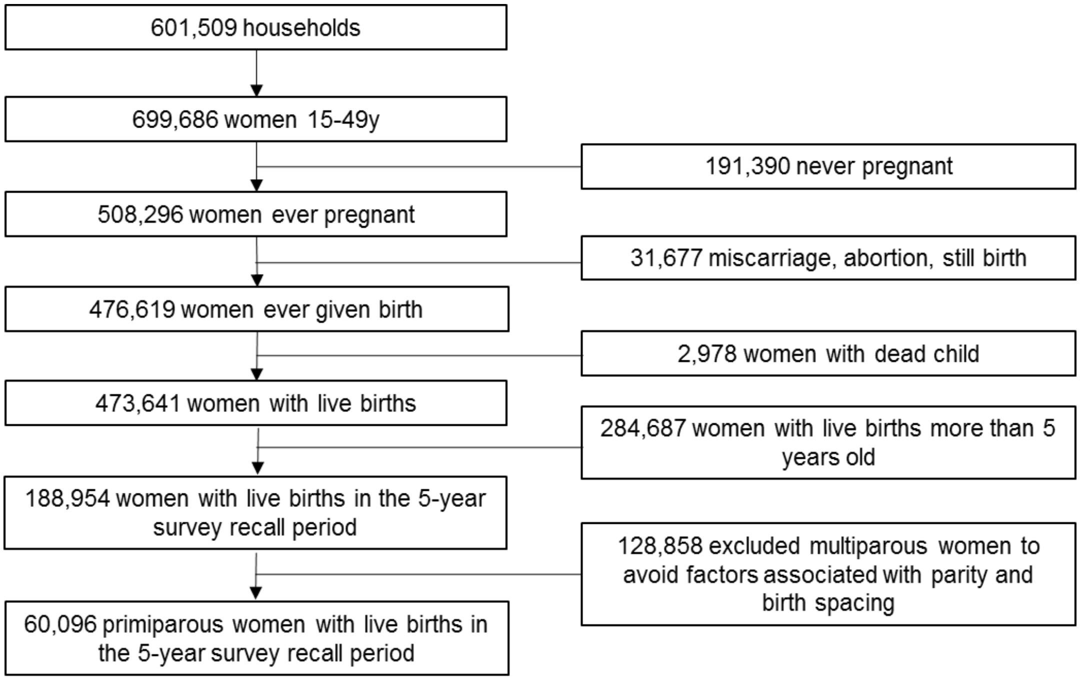

Supplemental Figure 2. Attained WAZ and underweight for first-born children by child age and mother's age at first birth in India, 2016.

A. Weight-for-age z-score

B. Underweight

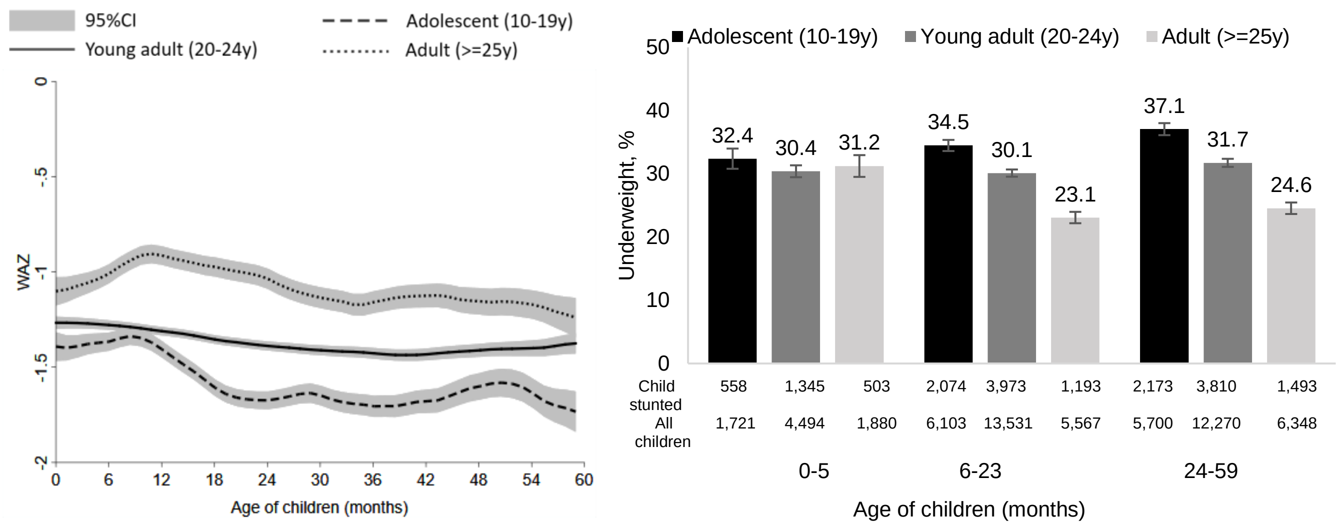

	OLS regression: WAZ		OLS regression: Underweight	
	β [95% CI]	*p*	*β [95% CI]*	*p*
Adolescence vs. young adulthood	-0·16 [-0·22,-0·09]	<0·0001	0·04 [0·02,0·06]	0·002
Adolescence vs. adulthood	-0·40 [-0·47,-0·32]	<0·0001	0·10 [0·07,0·12]	<0·0001

Curves are smooth local polynomials with 95% confidence intervals shown in gray. Adjusted coefficient [95% CI] for models with and without state fixed effects are shown below the figure. WHZ: weight-for-height z-score. Data are from India's fourth National Family Health Survey (NFHS4, 2016).

Supplemental Figure 3. Attained WHZ and wasting for first-born children by child age and mother's age at first birth in India, 2016.

A. Weight-for-height z-score

B. Wasting

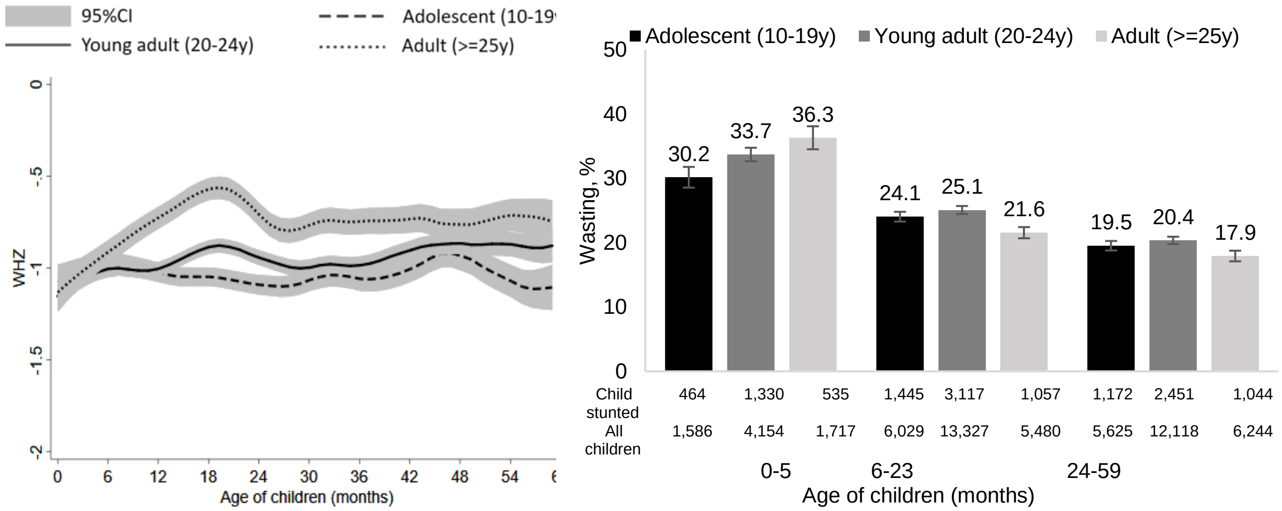

	OLS regression: WHZ		OLS regression: Wasting	
	β [95% CI]	*p*	*β [95% CI]*	*p*
Adolescence vs. young adulthood	-0·03 [-0·09,0·03]	0·298	-0·01 [-0·03,0·00]	0·033
Adolescence vs. adulthood	-0·16 [-0·23,-0·08]	<0·0001	0·01 [-0·01,0·02]	0·681

Curves are smooth local polynomials with 95% confidence intervals shown in gray. Adjusted coefficient [95% CI] for models with and without state fixed effects are shown below the figure. WHZ: weight-for-height z-score· Data are from India's fourth National Family Health Survey (NFHS4, 2016).

Supplemental Table 3. Comparing household, maternal and child characteristics of primiparous Indian women aged 15-49 years by sample with and without bargaining variables.

	Sample without bargaining variables (n=49,535)		Sample with bargaining variables (n=10,561)		p
**Household**	*Total women*	*N (%) or mean*	*Total women*	*N (%) or mean*	
Household size, n	49,535	5·89	10,561	5·64	<0·0001
Place of residence (rural)	49,535	35,329 (65·72)	10,561	7,322 (63·48)	0·053
Religion, %					
Hindu	49,535	36,932 (80·00)	10,561	7,818 (80·00)	1·000
Muslim	49,535	6,493 (13·95)	10,561	1,518 (14·38)	0·541
Other	49,535	6,110 (6·06)	10,561	1,225 (5·62)	0·325
**Mother**					
Age, y	49,535	24·05	10,561	24·18	0·079
Age at marriage, y	48,361	19·88	10,340	20·06	0·005
Age at first birth, y	49,535	22·14	10,561	22·26	0·071
Caste category, %					
Scheduled caste	49,535	8,606 (20·55)	10,561	1,793 (18·65)	0·006
Scheduled tribe	49,535	9,001 (9·44)	10,561	1,931 (9·60)	0·737
Other backward class	49,535	18,956 (41·58)	10,561	3,932 (42·37)	0·387
**Child**					
Age, mo	48,958	22·94	10,439	23·00	0·833
Male child, %	49,535	26,358 (53·60)	10,561	5,587 (53·34)	0·061

Data are from India's fourth National Family Health Survey (NFHS4, 2016).
